# At It Again

**Published:** 1898-04

**Authors:** 


### AT IT AGAIN.

Periodically some veterinarian or physician in Germany is suddenly attacked with a severe case of hysterics. The malady takes on various forms, according to the conditions at hand. If there is a piece of American meat anywhere near, harrowing shrieks are uttered to the public, denouncing the Yankee products as injurious to health. The latest case of hysterics has resulted in an article in the February number of the *Zeitschrift für Fleisch und Milch-hygiene*, and was superinduced by the finding of some nodules in American sausage-casings. Ignorant of the literature, the author pronounces the casings tubercular, and bewails the fact that Germany subjects herself and her inhabitants to the dangers alleged to be connected with importing American products. The intestines in question are of bovine origin, and the account of the lesions is an excellent description of a parasitic disease which is not transmissible to man. If our German confrérè will look up the published accounts of *Oesophagostoma columbianum*, he will probably change his diagnosis.

---

AN instructive commentary on the trichina question in Germany has just been published by Prof. Ostertag. A trichina inspector thought he found a peculiar parasite in one of his preparations; the supervising microscopist examined the slide and agreed that the structure in question was a parasite, but was unable to determine it. The preparation was then sent to one of the students of the Berliner Tierärztliche Hockschule for his opinion, and was finally submitted to Professor Ostertag for definite diagnosis. Ostertag recognized the alleged parasite as a "piece of hair!"

---

THE financial difficulties that threatened the progress of the work of the Pennsylvania State Live-stock Sanitary Board did not materialize, and this work is progressing as before. The storm of indignation that arose and was voiced by the press of the whole State when it was suggested that the board might be obliged to discontinue operations for a time constitutes an encouraging and hopeful indication as to the drift of public opinion on veterinary sanitation.

